# Immunotherapy in Non-Small Cell Lung Cancer Patients with Brain Metastases: Clinical Challenges and Future Directions

**DOI:** 10.3390/cancers13143407

**Published:** 2021-07-07

**Authors:** Ranjan Pathak, Arya Amini, Addie Hill, Erminia Massarelli, Ravi Salgia

**Affiliations:** 1 Department of Medical Oncology and Therapeutics Research, City of Hope, Duarte, CA 91010, USA; ahill@coh.org (A.H.); emassarelli@coh.org (E.M.); rsalgia@coh.org (R.S.); 2 Department of Radiation Oncology, City of Hope, Duarte, CA 91010, USA; aamini@coh.org

**Keywords:** non-small cell lung cancer, brain metastasis, PD-1, PD-L1, immunotherapy, immune checkpoint inhibitors

## Abstract

**Simple Summary:**

Although brain metastasis is a common and serious complication in lung cancers, current therapies with surgery, radiation, and traditional systemic agents are insufficient. Recently, immunotherapy has been shown to be effective against brain metastases in lung cancers, paving the way for a newer generation of systemic therapies. This review attempts to summarize our current understanding of immunotherapy in lung cancer brain metastases and explores future directions for research.

**Abstract:**

Immune checkpoint inhibitors have revolutionized the treatment landscape for patients with non-small cell lung cancers. Existing treatment paradigms for brain metastases in lung cancer patients leave patients with adverse neurocognitive function, poor quality of life, and dismal prognosis, thus highlighting the need to develop more effective systemic therapies. Although data are limited, emerging knowledge suggests promising activity and safety of immune checkpoint inhibitors in brain metastases in non-small cell lung cancer patients. This review aims to summarize the current data, highlight the challenges of incorporating immune checkpoint inhibitors in treating these patients, and identify areas for future research.

## 1. Introduction

Brain metastasis (BM) is a common and grave complication in non-small cell lung cancer (NSCLC). Almost a third of NSCLC patients develop brain metastasis at some point during their disease course, with higher rates reported in patients with epidermal growth factor receptor (EGFR) and anaplastic lymphoma kinase (ALK) mutations [1,2,3]. BMs are associated with adverse neurocognitive function, poor quality of life, and dismal prognosis despite multidisciplinary treatment with surgery, radiation therapy (RT), and systemic agents [4]. There is, therefore, a critical need for more effective therapies for NSCLC patients with BMs.

Over the past few decades, understanding of tumor biology and the immune system has led to the development of immune checkpoint inhibitors (ICIs) that have revolutionized the treatment landscape for patients with advanced NSCLC. Blocking the programmed death protein-1 (PD-1), its ligand (PD-L1), and cytotoxic T-lymphocyte-associated protein 4 (CTLA-4) pathways has led to remarkable improvements in the outcomes of these patients.

There are, however, limited data on the central nervous system (CNS) efficacy of ICIs, as most of the pivotal trials on ICIs excluded or underrepresented patients with BMs [5]. Some of the reasons for excluding these patients include concerns about the ability of monoclonal antibodies such as ICIs to penetrate the blood–brain barrier, diminished efficacy of ICIs due to concurrent steroid use, and hyperprogression of BMs [6,7]. Despite these concerns, several retrospective studies and prospective trials point toward the activity and safety of ICIs in NSCLC patients with BMs.

In this review, we aim to summarize the current clinical evidence for the efficacy of ICIs in NSCLC patients with BMs, highlight the challenges of incorporating ICIs in treating these patients, and identify areas for future research.

## 2. BM Inflammatory Microenvironment and Rationale for ICI-Based Treatment

Recent studies evaluating the immune system and tumor microenvironment (TME) have challenged the long-held view of the brain as an immune-isolated compartment [8]. It is now known that the brain parenchyma is an immunologically active organ that initiates and regulates immune responses [9]. The inflammatory TME of BMs consists of the innate immune system, namely microglia and blood-derived myeloid cells/macrophages, and the adaptive immune system, mainly represented by T cells [10]. Varying degrees of T cell infiltration or tumor-infiltrating lymphocytes (TILs) have been observed in BMs [11,12]. Patients with BMs with dense infiltration of effector CD3+, cytotoxic CD8+, or memory CD45RO+ TILs have been found to have improved survival compared with patients with low or absent TILs [13]. Unlike cytotoxic chemotherapy and targeted therapies, because ICIs act by removing the inhibition of T cells by tumor cells, immune cell trafficking of peripherally activated T cells into the CNS is perhaps more critical than the penetration of the blood–brain barrier by the ICIs themselves [9]. However, recent observations have suggested that cerebrospinal fluid concentrations of systemically administered pembrolizumab (which were 1% of serum concentrations) can functionally block PD-1 on T cells [14]. These observations, along with the recent discovery of the CNS lymphatic system and TME preconditions in the CNS that mimic extracranial metastases [15,16]] are challenging our long-held notions of immune privilege in the CNS and support the clinical development of ICI-based strategies in patients with BMs.

## 3. Safety and Efficacy of ICIs in Patients with NSCLC BMs

Currently, there are limited prospective data on the efficacy and safety of ICIs in NSCLC patients with BMs. Patients with BMs have historically been underrepresented in the clinical trials of ICIs in NSCLC. For example, a study by El Rassy et al. found that only 6.2–17.5% of the patients enrolled in the pivotal NSCLC trials had asymptomatic or previously treated and stable BMs, and none of them allowed patients with symptomatic or untreated BMs [5]. Besides, the majority of these trials do not report intracranial efficacy and other outcomes stratified by the presence or absence of BMs. As a result, our current understanding of the efficacy and safety of ICIs in BMs in NSCLC have primarily been derived from the single-arm phase 1–2 trials [17,18], expanded access programs [19,20,21], post hoc/pooled analyses of clinical trials [22,23,24,25,26,27], and retrospective series [28,29,30,31,32,33,34,35].

### 3.1. Untreated BMs

Given the concerns for increasing peritumor inflammation and vasogenic edema, the majority of ICI trials have excluded NSCLC patients with BMs that have not received local therapies such as RT. However, recent data presented below challenge this notion and provide evidence that ICI therapy by itself might be able to achieve intracranial response with acceptable safety in a select group of patients. 

#### 3.1.1. ICI Monotherapy

Goldberg et al. have recently reported an updated analysis of the NSCLC cohort of their phase 2 trial of pembrolizumab in patients with NSCLC or melanoma with untreated brain metastases [18]. Forty-two patients with stage IV non-oncogene driven, ICI-naïve NSCLC with at least one asymptomatic BM measuring 5–20 mm in size, not previously treated or progressing after previous RT, and not requiring steroids, were treated with pembrolizumab 10 mg/kg intravenously every 2 weeks. After a median follow-up of 8.3 months, 11 (29.7%, 95% CI, 15.9–47.0) of 37 patients in cohort 1 showed intracranial response with a CNS progression-free survival (PFS) of 2.3 months (95% CI, 1.9-not reached) with almost one-third of patients remaining progression-free in the CNS at 1 year. Thirty-four percent (95% CI 21–54) of the patients were alive at 2 years. No responses were seen in the PD-L1 negative patients (5 patients in cohort 2). Treatment-related serious adverse events occurred in 6 (14%) of 42 patients and were comparable with adverse events reported in other ICI trials. No treatment-related neurological adverse events were noted. This study provides a prospective proof of concept for the intracranial activity of ICI in patients with NSCLC (Table 1). 

Another prospective trial that specifically evaluated NSCLC patients with untreated BMs was the Checkmate 012 [17]. Patients in “arm M” of this phase 1 trial included 12 patients with at least one asymptomatic and untreated BM up to 30 mm in size. Patients were required to have at least one prior systemic therapy for NSCLC and could have up to four BMs. Two intracranial responses were observed (16.7%, 95% CI, 2.1–48.4) and the median PFS was 1.6 months (95% CI 0.92–2.50) with a median overall survival (OS) of 8.0 months (95% CI, 1.38–15.50). Similarly to the study by Goldberg et al., no treatment-related neurologic adverse events were reported.

Several other ongoing single-arm phase 2 trials are evaluating the role of ICI in patients with untreated BMs (Clinicaltrials.gov NCT02681549, NCT02886585, NCT03526900) (Table 2). The intracranial efficacy will be measured by modified RECIST in the first study, while Response Assessment in Neuro-Oncology Brain Metastases (RANO-BM) criteria will be used for the other two studies [36]. 

In addition to these prospective trials, several other retrospective studies have suggested the potential efficacy of ICI alone in untreated BMs and are summarized in Table 1. However, the patients in the above studies were highly selected and only included patients that had small BMs and were asymptomatic. Therefore, further studies are needed to better clarify the efficacy and safety of ICI alone for untreated BMs that are larger or are symptomatic.

#### 3.1.2. Concurrent ICI and RT

The addition of RT to ICI has been investigated as a means to create synergy between the two treatment modalities by priming the immune response and, possibly, an abscopal response (tumor regression at a site distant from the primary site of radiotherapy) [37,38]. Although RT can lead to leukopenia and cause immunosuppression, it can also stimulate the innate and adaptive immune system through the release of tumor cell antigens and activation of critical molecular pathways, including increased PD-L1 expression [39,40].

Several studies, including a meta-analysis of retrospective data on stereotactic radiosurgery (SRS) and ICI, have suggested better OS with concurrent rather than sequential ICI and SRS [41,42]. Ahmed et al. reported retrospective data on NSCLC BM patients who received SRS and anti-PD-1/PD-L1 therapy [43]. Patients who received RT during or before ICI therapy achieved better distant brain control rate at 6 months compared with patients who received ICI before RT. The rate of acute neurotoxicity was similar among patients who received SRS alone or with ICI (ICI was given either within 2 weeks of SRS or afterward). Grade 3 acute CNS toxicity was similar between the two interventions.

Discussion on the optimal timing and dosing of RT with ICI was outside the scope of this review, and the results of ongoing clinical trials of RT in patients with BMs are expected to give more insight into this important clinical question (such as NCT02696993, NCT02858869, NCT02710253).

### 3.2. Pretreated BMs

#### 3.2.1. ICI Monotherapy

Several post hoc analyses of ICIs in NSCLC patients with pretreated, asymptomatic, and stable BMs have been reported in recent years [18,24,25]. In a pooled analysis of NSCLC patients with BMs enrolled in three trials with nivolumab (CheckMate 063, 017, and 057), 46 patients who received nivolumab as second-line treatment displayed acceptable safety and promising efficacy when compared to docetaxel [24]. Similarly, in another pooled analysis of pembrolizumab monotherapy trials (KEYNOTE-001, 010, 024 and 042), pembrolizumab showed improved survival with pembrolizumab compared with chemotherapy, irrespective of BM at baseline [22].

#### 3.2.2. ICIs Combined with Chemotherapy

The combination of ICIs with chemotherapy represents another recent advance in the treatment of advanced NSCLC patients, with multiple front-line trials showing the combination to be superior to chemotherapy alone [44]. A pooled analysis of pembrolizumab plus chemotherapy trials (KEYNOTE-021, 189, and 407) has shown the combination to improve survival irrespective of the presence or absence of BM at baseline (Table 3) [23].

#### 3.2.3. Dual ICI Therapy

Borghaei et al. recently presented a post hoc analysis of the BM-positive cohort from the Checkmate 227 trial that randomized advanced NSCLC patients into first-line ipilimumab plus nivolumab versus chemotherapy [27]. The data suggested similar efficacy and safety of dual-ICI therapy for NSCLC patients irrespective of the presence or absence of BMs at baseline [27] (Table 3).

## 4. Current Clinical Challenges

Despite the favorable evidence suggesting that ICIs can provide intracranial responses in patients with untreated or treated BMs, several questions remain about the optimal use of ICIs in these patients, as discussed below.

### 4.1. New Untreated (Systemic Therapy Naïve) BMs

#### 4.1.1. Small Asymptomatic BMs

Limited evidence, as discussed earlier, provides proof of principle that ICIs can induce objective intracranial responses in patients with BMs [17,18]. Preliminary data have shown durable responses that were concordant with extracranial response in most patients (almost 80%) with NSCLC BMs, with intracranial ORRs approaching 30% [18]. These data suggest that ICI might be an acceptable treatment option for select patients eligible for ICI with small (perhaps <2 cm), asymptomatic or minimally symptomatic, previously untreated brain metastasis. Patients treated with upfront ICI with deferred local therapy require careful clinical and imaging monitoring to detect CNS progression. For patients that are treated with upfront ICI and deferred local CNS therapy, short-interval imaging should be considered, which can be performed with contrast-enhanced magnetic resonance imaging (MRI) (or contrast-enhanced computed tomography if MRI is not possible) every 4–6 weeks in the beginning, followed by every 2 to 3 months.

#### 4.1.2. Large or Symptomatic BMs

For patients with symptomatic BMs or multiple large BMs, rapid local control is crucial, as the progression of BMs can lead to rapid functional deterioration, impaired quality of life, or possibly death [46]. These patients often require a course of steroids to manage CNS symptoms in conjunction with upfront local CNS therapy in collaboration with a multidisciplinary team consisting of radiation oncologists and neurosurgeons [47]. Systemic treatment with ICI should be paused until steroids are tapered to a safe level given concerns of inferior efficacy of ICI with steroids [48]. Although there are no well-established data or thresholds for steroid use in this context, we suggest trying to taper dexamethasone to a dose of 2 mg twice daily or less before initiation of ICIs.

### 4.2. Treated BMs

ICI therapies are acceptable treatment options for patients with treated NSCLC BMs given pooled data from numerous clinical trials of ICI monotherapy, ICI dual therapy, or ICI-chemotherapy showing similar survival outcomes in patients with or without baseline BMs [22,23,24,25,27].

### 4.3. New BMs in Patients Receiving ICIs

For patients who develop brain metastases as a site of progression during or after ICI, options for systemic therapy are limited, and control of CNS disease is often best achieved with either surgery and/or RT, with the choice and sequence of definitive CNS therapy depending on the number, size, and location of BMs, the extent of CNS symptoms, and overall performance status [47]. If BM represents the only site of disease progression, then surgical resection of an isolated metastasis with the continuation of ICI-based systemic therapy might be an option. However, most patients who develop progression of BMs on ICI will require local treatment along with a change in systemic therapy to subsequent line systemic therapy or clinical trials.

## 5. Special Considerations in the Treatment of BM with ICIs

### 5.1. Timing of ICI with RT

Despite concerns for increased risk of radiation necrosis, there is sparse data on the optimal timing and sequencing of RT and ICI in patients who require RT for symptomatic BMs [41]. Most retrospective studies have shown a manageable short-term safety profile for patients receiving intracranial RT concurrently for BMs while on ICI for extracranial disease [49].

### 5.2. Risk of Radiation Necrosis with ICI and RT

Radiation necrosis or treatment-induced brain tissue necrosis is a critical delayed complication of radiation therapy that usually develops 6 months to 2 years after radiation [50]. Radiation necrosis is thought to be more common with higher doses per fractionation and with concurrent chemotherapy or radiosensitizers, [51] and is thought to be more frequent with SRS, especially in the setting of concurrent administration of ICIs [52,53]. Radiation necrosis is difficult to distinguish from tumor recurrence radiographically and often requires biopsy or serial imaging, as radiation necrosis tends to regress spontaneously after an initial period of growth.

Development of radiation necrosis in patients receiving ICI can be challenging as these patients often require moderate to high doses of steroids that can potentially lower both intracranial and extracranial efficacy of ICIs [54]. In these scenarios, bevacizumab and surgery can be used selectively to control symptoms and facilitate steroid taper [55,56].

### 5.3. Leptomeningeal Disease

Although ICI may be considered in this setting, data on ICI are extremely limited. For patients with increased intracranial pressure or hydrocephalus related to ICI, insertion of ventriculoperitoneal shunt should be considered to control intracranial pressure and allow time for ICI response. As in the case with BMs, in the absence of prospective data, it is difficult to draw any definitive conclusions regarding the optimal timing and sequencing of whole brain RT with ICIs.

### 5.4. Pseudoprogression

Pseudoprogression involves a transient enlargement of existing lesions or the appearance of new lesions mimicking tumor progression, which resolves on longitudinal imaging [57]. ICIs (particularly anti-CTLA-4 agents) have been known to result in pseudoprogression when used to treat BMs [58]. Imaging studies in patients with pseudoprogression often show an increased contrast enhancement and vasogenic edema (which can be small and asymptomatic), which usually occur within the first 3 months. These changes usually stabilize or subside on further follow-up without additional therapies. For minimally symptomatic lesions, close follow-up with serial imaging can avoid unnecessary tumor-directed therapies. Sometimes, a biopsy is needed to distinguish treatment-related changes from progressive tumors and to guide further therapy [58].

### 5.5. CNS Toxicities with ICI Therapy for BMs

Evaluating the actual clinical impact of neurologic adverse events in BM patients treated with ICIs is difficult given issues with assessing the adverse events as being tumor-associated inflammatory response, paraneoplastic, or truly autoimmune events, and variable reporting of neurotoxicity across trials [59]. The majority of data for ICI-related neurotoxicity in BM patients come from trials conducted in melanoma patients. CNS autoimmune toxicities due to ICIs are rare but can include myasthenia gravis, encephalitis, aseptic meningitis, and rarely, multiple sclerosis. Available studies on the use of ICIs in NSCLC BMs so far have reported a very low incidence of autoimmune CNS toxicities in these patients [59].

### 5.6. Future Risk of New CNS BM Development

In the OAK trial of subsequent line atezolizumab versus docetaxel, the risk of identifying new symptomatic brain lesions (patients were not required to undergo regularly scheduled follow-up scans and were instead symptom-driven) in patients with a history of asymptomatic, treated BM appeared to be lower than with docetaxel. In patients without BM at baseline, larger sample size and a longer follow-up period are needed to generate enough data to draw any meaningful conclusion regarding the future risk of BMs in patients treated with atezolizumab [26]. Similar results were seen in the pooled analysis of five studies (PCD4989g, BIRCH, FIR, POPLAR, and OAK) that evaluated subsequent-line atezolizumab versus docetaxel, with a lower risk of developing a new CNS lesion with atezolizumab (median time to develop a new CNS lesion, not reached versus 9.5 months; HR 0.42, 95% CI, 0.15–1.18) [25].

Post hoc analysis of data from the Impower 150 trial showed that the combination of atezolizumab plus bevacizumab plus carboplatin plus paclitaxel (ABCP) might delay the time to new BM development compared with atezolizumab plus carboplatin plus paclitaxel (ACP) [60]. Authors reported that with a minimum follow-up of 32.4 months (with the development of BM in 100 patients), ABCP regimen was associated with a lower rate of new BM development (7.0%) compared with ACP (11.9%) and BCB (6.0%) regimens. Median time to develop new BMs was not reached in any arms; however, a trend toward delayed time to new BM development was seen in the ABCP arm versus the BCP arm (HR 0.68; 95% CI, 0.39–1.19). The rates of grade 3–4 treatment-related adverse events were similar between the patients with and without BMs but were slightly higher in the ABCP arm than the ACP and BCP arms [60].

## 6. Conclusions and Future Directions

Although only a few prospective ICI trials have included NSCLC patients with untreated BMs, preliminary results show encouraging intracranial activity of ICIs that are comparable to extracranial responses. Pooled analyses of the trials indicate that patients with BMs achieve similar OS compared to those without BMs. However, because only a highly select group of patients with previously treated or asymptomatic BMs have been treated in clinical trials so far, the broader applicability of these results to NSCLC patients is limited. Even though the PD-L1 tumor proportion score has been used as a predictive biomarker in the treatment of advanced NSCLC, it is unclear whether PD-L1 expression in extracranial lesions can be used to select BM patients for ICIs as some studies have suggested a low level of concordance in PD-L1 expression between primary and metastatic sites [61]. As it can be challenging to obtain tissues from the BMs to carry out PD-L1 testing, further studies are needed to clarify whether PD-L1 expression in BMs is needed for the intracranial benefit with ICIs. Some of the other areas that need to be explored include the risks of vasogenic edema and pseudoprogression with ICIs and the timing/sequencing of RT and ICIs. Finally, refinement in the intracranial disease response criteria and definitions of measurable disease are needed to standardize clinical trial conduct in patients with BMs.

NSCLC patients, including those with BMs, are living longer with the introduction of ICIs. Early evidence suggests acceptable safety and promising efficacy of ICIs even in patients with untreated BMs. Additional clinical trials and translational studies are needed to expand and refine the role of ICI in NSCLC patients with BMs.

## Figures and Tables

**Table 1:** Summary of clinical studies with immune checkpoint inhibitors in patients with brain metastases.

Author, Year	Trial	Phase	LOT	N	Histology	PD-L1	CNS Disease	ICI Arm	Comparator Arm, If Present	F/u	CNS ORR	Median CNS PFS	Extracranial ORR	DOR	Median PFS (mo) or PFS HR	Median OS (mo) or OS HR		Notes
Mansfield, 2019 [22]	Pooled Analysis of KEYNOTE-001, −010, −024, and −042	Ib to III	≥1	293/3170	Squamous + non-squamous	PD-L1 TPS ≥1%	Treated and stable	Pembro	Chemo	18.4	-	-	26.1% (20.2–32.8) vs. 25.8% (23.7–27.9)	NR (IQR 3.3 to 46.2+) vs. 30.4 (IQR 1.4+ to 49.3+)	0.96 (0.73–1.25) vs. 0.91 (0.84–0.99)	13.4 vs. 10.3; 0.83 (0.62–1.10) [vs. 14.8 vs. 11.3; 0.78 (0.71–0.85)]		TRAEs occurred similarly with pembro vs. chemo both in pts with BM (66% vs. 84%) and without (67% vs. 88%)
Goldberg, 2020 [18]	NCT02085070	II	≥1	42	Squamous + non-squamous	PD-L1 TPS ≥1% (*n* = 37) or 0% (*n* = 5)	Untreated and asymptomatic (5 mm to 20 mm)	Pembro	-	8.3	29.7% (15.9–47.0)	2.3 (1.9-not reached)	-	6.9 (IQR 3.7–22.4)	1.9 (1.8–3.7)	9.9 (7.5–29.8)		6/27 patients had discordant response.
Goldman, 2016 [24]	Pooled Analysis of Checkmate 063, 017 and 057	II to III	≥2	46	Squamous + non-squamous	NA	Treated and stable	Nivo	Docetaxel	8.4	-	-	-	-	-	Checkmate 017: 4.99 vs. 3.86 (nivo vs. docetaxel) (HR not reported); Checkmate 057: 7.61 vs. 7.33; 1.04 (0.62–1.76)		CNS TRAEs occurred in 5 pts (11%) and were all gr 1–2 (paresthesia, *n* = 2; dizziness, somnolence, and tremor, *n* = 1 each)
Hellman, 2017 [17]	Checkmate 012, Arm M	I	≥2	12	-	-	Untreated and asymptomatic (≤3 cm and ≤4 in number)	Nivo	-	-	16.7 (2.1–48.4)	-	-	-	1.6 (0.92–2.50)	8.0 (1.38–15.50)		2 out of 12 patients achieved intracranial responses, including a patient with leptomeningeal disease
Lukas, 2017 [25]	Pooled analysis of PCD4989g, BIRCH, FIR, POPLAR, and OAK	I to III	≥2	79/1452	Squamous + non-squamous	Unselected	Treated and stable	Atezo	Chemo	-	-	-	-	-	-	20.1 vs. 11.9; 0.54 (0.31–0.94) vs. 13.0 vs. 9.4; 0.75 (0.63–0.89)		Incidence of all AEs and SAEs was similar in pts with or without BMs. The most common treatment + R8-related neurological AE was headache in 6 (8%) pts with and 42 (3%) pts without BM.
Gagdeel, 2018 [26]	OAK	III	≥2	123/850	Squamous + non-squamous	Unselected	Treated and stable	Atezo	Docetaxel	28	-	Time to radiographic identification of new symptomatic BM: NR vs. 9.5; 0.38 (0.16–0.91)	-	-	-	16.0 vs. 11.9; 0.74 (0.49–1.13) [vs. 13.2 vs. 9.3; 0.74 (0.63–0.88)]		No treatment-related grade 4–5 neurologic AEs or SAEs were observed in patients with a history of asymptomatic, treated BM, and there was a low incidence of treatment-related grade 3 neurologic AEs (5%).
Hendriks, 2019 [28]	Retrospective study that included patients on routine clinical care, EAPs, compassionate use programs, and clinical trials		1	255/1025	Squamous + non-squamous	Unselected	Untreated and asymptomatic or treated and stable (stable or decreasing symptoms allowed)	Anti-PD-1 or anti-PD-L1 monotherapy	-	15.8	27.3 (PD-L1 positive patients (*n* = 14): 35.7%; PD-L1 negative (*n* = 9): 11.1%)	-	20.6% vs. 22.7%	-		1.7 (1.5–2.1) vs. 2.1 (1.9–2.5) (with and without BM)	8.6 (6.8–12.0) vs. 11.0 (8.6–13.8)	Multivariable analysis showed that steroid use (HR, 2.37) was associated with poorer OS, whereas stable BMs (HR, 0.62) and higher ds-GPA classification (HR, 0.48–0.52) were associated with improved OS.
Crino, 2017 [19]	Retrospective (Italian EAP)		≥2	409/1588	Non-squamous	-	Asymptomatic	Nivo	-	6.1	-	-	-	-		-	-	-
Molinier, 2017 [20]	Retrospective (French EAP)		≥2	130/600	Squamous + non-squamous	Unselected	NR	Nivo			12% partial response						6.6	7 patients had all-grade neurological symptoms, 1 (0.1%) grade 3, not specified whether it was BM patient or not.
Gauvain, 2018 [29]	Retrospective		≥2	43/191	Squamous + non-squamous	unselected	Included all patients whether treated or not, symptomatic or asymptomatic	Nivo	-	5.8	9.0 (3.0–23.0)	3.9 (2.8–11.1)	11% (4–26)	-		-	-	Five neurological events occurred, including 1 grade-4 transient ischemic attack of uncertain imputability and 1 grade-3 neurological deficit; neither required nivo discontinuation.
Cortinovis, 2017 [21]	Retrospective (EAP Italy)		≥2	38/372	Squamous	unselected	Treated and stable	Nivo	-	4.5	-	-	-	-		5.5	6.5	Disease control rate was 47.3%, including 1 complete response, 6 partial responses, and 11 stable diseases. Out of the 38 patients included, only 1 discontinued treatment due to AE (2.6%), whereas 21 pts (55.3%) discontinued treatment for non-toxicity related reasons.
Watanabe, 2017 [30]	Retrospective		≥2	4 out of 48	-	-	Untreated	Nivo	-	-	-	-	-	-		1.8	-	None of the BM patients treated with nivolumab achieved intracranial response.
Dudnik, 2016 [31]	Retrospective		≥2	5	Squamous + non-squamous	NA	Untreated but asymptomatic	Nivo	-	-	-	-	-	-		-	-	Two intracranial responses were observed, including one complete response of parenchymal brain metastases and one partial response of leptomeningeal carcinomatosis. All of the responses were rapid and durable. Importantly, no grade 3/4 adverse events were seen. Systemic responses and intracranial responses were largely concordant
Bjørnhart, 2019 [32]	Retrospective		-	21	-	-	-	Nivo or pembro	-	-	4.8	-	-	-		4.2 (2.5–5.9)	8.2 (1.0–15.5)	-
Dumenil, 2018 [33]	Retrospective		-	10	-	-		Nivo	-	-	-	-	-	-		-	3.1	-
Garde-Noguera, 2018 [34]	Retrospective		-	38	-	-		Nivo	-	-	-	-	17.2	-		1.6	3.1	-
Sun, 2020 [35]	Retrospective		≥1	66	Squamous + non-squamous		Treated and stable or received RT within 30 days of starting pembro or untreated	-	-	15	-	-	-	-		9.0 vs. 7.9 (with or without BM)	18.0 vs. 21.0 (with or without BMs)	13 treated with pembro alone, intracranial responses included 2 CR, 2 PR, 3 SD, and 4 PD. On multivariable analysis, female sex, ECOG 0–1, adenocarcinoma histology, and P as first line therapy were associated with improved PFS and OS. Presence of BM, baseline steroid use, and timing of local RT (before vs. after P) were not associated with inferior survival

Atezo = atezolizumab; BM = brain metastasis; chemo; CNS = central nervous system; CR = complete response; DOR = duration of response; ECOG = Eastern Cooperative Oncology Group; EAP = expanded access program; F/u = median follow up in months; ICI = immune checkpoint inhibitor; ipi = ipilimumab; LOT = line of therapy; mo = months; N = number of patients with brain metastasis; nivo = nivolumab; NR = not reported; OS = overall survival; pembro = pembrolizumab; PD = progression of disease; PD-L1 = Programmed death-ligand 1; PFS = progression free survival; PR = partial response; RT = radiation therapy; SD = stable disease.

**Table 2:** Ongoing clinical trials with immune checkpoint inhibitors in patients with untreated brain metastases.

Clinicaltrials.Gov Identifier	Phase	Disease	Major Inclusion Criteria	Steroids	Intervention	Estimated Enrollment
NCT02681549	II	NSCLC and melanoma	At least one untreated BM 5–20 mm, asymptomatic, and not requiring steroids, PD-L1 positive	Steroids not permitted	Pembrolizumab plus bevacizumab	53
NCT02886585	II	NSCLC and melanoma	Untreated asymptomatic BM or progressive asymptomatic BM measuring ≥10 mm or cytology positive neoplastic meningitis	Stable dose of dexamethasone 2 mg/day or less for 7 days prior to initiation of treatment	Pembrolizumab	102
NCT03526900	II	NSCLC	Untreated BM, asymptomatic, and ≤4 mg dexamethasone/day	Up to ≤4 mg dexamethasone/day allowed as long as patients are asymptomatic or minimally symptomatic	Atezolizumab plus carboplatin plus pemetrexed, followed by maintenance atezolizumab plus pemetrexed	40

BM = brain metastasis; NSCLC = non-small cell lung cancer; PD-L1 = programmed death-ligand 1.

**Table 3:** Summary of clinical studies with immune checkpoint inhibitor combinations in patients with brain metastases.

Author, Year	Trial	Phase	LOT	N	Histology	PD-L1	CNS Disease	ICI Arm	Comparator Arm, If Present	F/u	Extracranial ORR, %	DOR, mo	Median PFS (mo) or HR for PFS	Median OS (mo) or HR for OS	Notes
**Chemoimmunotherapy**															
Powell, 2019 [23]	Pooled analysis of KEYNOTE-021, 189, and 407	II, III	1	171/1298	Squamous + non-squamous	Unselected	Treated and stable	Pembro + chemo	Chemo	10.9	39 vs. 19.7 [vs. 54.6 vs. 31.8]	11.3 vs. 6.8 [vs. 12.2 vs. 6.0]	6.9 vs. 4.1; 0.44 (0.31–0.62) [vs. 8.8 vs. 5.3; 0.55 (0.48–0.63)]	18.8 vs. 7.6; 0.48 (0.32–0.70) [vs. 22.5 vs. 13.5; 0.63 (0.53–0.75)]	All-cause grade 3–5 AEs with Pembro + chemo vs. chemo alone occurred in 81.4% vs. 70.3% of pts with BM and 68.3% vs. 65.6% without BM.
Afzal, 2018 [45]	Retrospective		≥1	18/54	Non-squamous	Unselected	Treated and stable	Pembro + chemo		30	80		6.5	13.7	-
**Dual ICI**															
Borghaei, 2020 [27]	Checkmate 227	III	1	135/1739	Squamous + non-squamous	Unselected	Treated and stable	Ipi + nivo	Chemo	29.3 (minimum follow-up)	33 vs. 26 [vs. 33 vs. 28]	24.9 (11.3–NR) vs. 8.4 (4.2–13.9) [vs. 19.6 (15.5–28.6) vs. 5.8 (4.8–6.9)]	5.4 vs. 5.8; 0.79 (0.52–1.19) 4.9 [vs. 5.4; 0.81 (0.70–0.93)]	18.8 vs. 13.7; 0.57 (0.38–0.85) [vs. 17.1 vs. 13.9; 0.76 (0.66–0.88)]	Any-grade nervous system adverse events were reported in 46% of pts with BM treated with ipi + nivo and 42% of those treated with chemo, most were grade 1–2.

BM = brain metastasis; CNS = central nervous system; DOR = duration of response; chemo; F/u = median follow up in months; ICI = immune checkpoint inhibitor; ipi = ipilimumab; LOT = line of therapy; mo = months; N = number of patients with brain metastasis; nivo = nivolumab; NR = not reported; OS = overall survival; pembro = pembrolizumab; PFS = progression free survival.

## Data Availability

No primary data reported.
